# A Multispecies Systematic and Critical Review of Intranasal Administration in Veterinary Anaesthesia and Emergency Care: Promising Evidence and Overlooked Challenges

**DOI:** 10.1002/vms3.71041

**Published:** 2026-06-18

**Authors:** Majid Jafarbeglou

**Affiliations:** ^1^ Independent Clinician and Researcher Tehran Iran

**Keywords:** anaesthesia, critical care, emergency, intranasal, veterinary

## Abstract

Intranasal (IN) drug delivery has increasingly considered as an easy, practical and non‐invasive alternative to parenteral administration in veterinary medicine, offering rapid systemic and potential nose‐to‐brain effects. The first part of this review systematically collected and synthesized published evidence on IN administration across animal species, while the second part critically analysed the anatomical, pharmacological and technical factors that determine its success and limitations. Part I consisted a total of 110 eligible studies published between 1991 and 2025, encompassing dogs, cats, rabbits, pigs, ruminants, birds and reptiles. IN delivery has been investigated for a range of purposes and produced clinically meaningful sedation, analgesia and drug reversal, often comparable to intramuscular administration but generally characterized by slower onset and greater variability among species. Despite encouraging and favourable results, IN delivery was not without limitations. Its effectiveness can be strongly influenced by species‐specific nasal anatomy and physiology, formulation characteristics and dosing volume. Defensive reactions, poor tolerability, sneezing, nasopharyngeal irritations, hypersalivation or swallowing of the drug are frequently reported. Future progress requires species‐specific case selection guidelines and dosing standards, pharmacokinetic validation and developing safe concentrated formulations. Transparent reporting and balanced assessment of both benefits and drawbacks are essential to ensure the safe, effective and ethically responsible integration of IN administration into veterinary anaesthesia and critical care practice.

## Highlights

- Intranasal (IN) delivery offers practical route in anaesthesia and critical care
- Nasal anatomy and physiology differences strongly influence IN absorption
- Technical and formulation factors often reduce efficacy and tolerability
- Nasal irritations, adverse behaviours and defensive reactions frequently reported
- IN delivery may be physiologically or behaviourally invasive in many cases

## Graphical abstract

This review evaluates the clinical potential and limitations of intranasal (IN) drug administration in veterinary anaesthesia and emergency care. IN delivery can provide clinically relevant sedation, analgesia and drug reversal, but its success is not universally reliable and is strongly influenced by species‐specific anatomy, formulation characteristics and administration techniques. Variable absorption, inconsistent clinical responses and tolerability issues are common, with swallowing, sneezing, defensive reactions and aggression among the most frequently reported complications. These findings highlight the need for standardized protocols and further validation to ensure safe, effective and ethical clinical application.

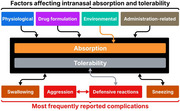

## 1 Introduction

Intranasal (IN) drug delivery has gained increasing attention in veterinary medicine as a convenient and non‐invasive alternative to parenteral administration (Araghi et al. 2016; Lin et al. 2024; Marjani et al. 2015). The thin, highly vascularized nasal epithelium provides a large absorptive surface that facilitates the rapid uptake of lipophilic, low‐molecular‐weight compounds while bypassing gastrointestinal degradation and hepatic first‐pass metabolism (Bustamante et al. 2024; Micieli, Santangelo, Reynaud, et al. 2017). In addition, direct ‘nose‐to‐brain’ transport via the olfactory and trigeminal nerves, as well as through cerebrospinal fluid pathways, can partially circumvent the blood–brain barrier (Jafarbeglou and Marjani 2019; López‐Ramis et al. 2022). These mechanisms are particularly advantageous for centrally acting agents and have generated growing interest in IN administration, especially in situations where intravenous (IV) access or intramuscular (IM) injection is impractical, stressful or unsafe (Charalambous et al. 2019; Hommuang et al. 2022; Santangelo et al. 2019). Reported applications include sedation and chemical restraint, anaesthetic induction, analgesia and pain management and emergency interventions such as seizure control, reversal of accidental exposure or overdose, anaphylaxis and cardiopulmonary resuscitation.

Despite encouraging results across species and drug classes, IN delivery is not without limitations. Absorption may be affected by dosing volume, formulation and interspecies or interindividual variability (Rabelo et al. 2024; Schnellbacher et al. 2012). Our own decade‐long clinical experience with this route in veterinary patients suggests that IN delivery is not free from complications, a perspective echoed in multiple studies. Defensive reactions, poor tolerability, sneezing, nasopharyngeal irritations, hypersalivation or swallowing of the drug are frequently reported, potentially reducing drug bioavailability and clinical efficacy (Breitenlechner et al. 2024; Bustamante et al. 2024; Jafarbeglou, Marjani, Bakhshi‐Khanghah, et al. 2024; Santangelo, Micieli, Mozzillo, et al. 2016; Vlerick et al. 2020).

To the author's knowledge, although several reviews exist in human medicine, no comprehensive review has yet addressed IN drug delivery in veterinary medicine. Therefore, the objective of Part I of this review is to systematically collect, organize and synthesize the scattered but steadily expanding evidence on IN administration across different animal species. Part II aims to critically evaluate the techniques used, and to explore whether, alongside its promising potential, there remain underexplored or overlooked limitations or ‘blind spots’ that could constrain the safe, consistent and reliable adoption of IN delivery in veterinary anaesthesia and critical care.

### 1.1 Search Strategy and Data Extraction

A comprehensive literature search was conducted in Google Scholar, PubMed, Scopus and ResearchGate up to 16 September 2025. The search strategy combined controlled vocabulary (e.g., MeSH terms) and free‐text keywords related to IN administration and veterinary applications. Search terms included ‘intranasal’, ‘nasal’, ‘transnasal’, ‘veterinary’, ‘sedation’, ‘analgesia’ ‘anesthesia’ and specific drug names combined with species names. Articles about IN administration in veterinary species reporting outcomes relevant to anaesthesia or emergency care were included. The search was supplemented by manual screening of the reference lists and citation networks of eligible articles to identify additional relevant publications not captured by database searches.

Exclusion criteria were non‐English publications without reliable translation, lack of full‐text access, studies on CNS‐acting drugs unrelated to veterinary anaesthesia or critical care (e.g., oxytocin), non‐CNS‐acting drugs (e.g., vaccines, antibiotics) and studies limited to local effects (e.g., decongestants, local anaesthetics). In addition, two publications on dogs and rabbits by the same authors from journals listed in Beall's list of potential predatory journals (beallslist.net/standalone‐journals) were excluded. Most rodent studies were conducted purely as experimental pharmaceutical models without translational relevance to veterinary anaesthesia or emergency medicine, and therefore omitted. Two Portuguese articles from Brazil with English abstract, as well as some shorter publications (case reports, conference abstracts and letters to the editor), were included because they contained valuable information.

**FIGURE 1:**
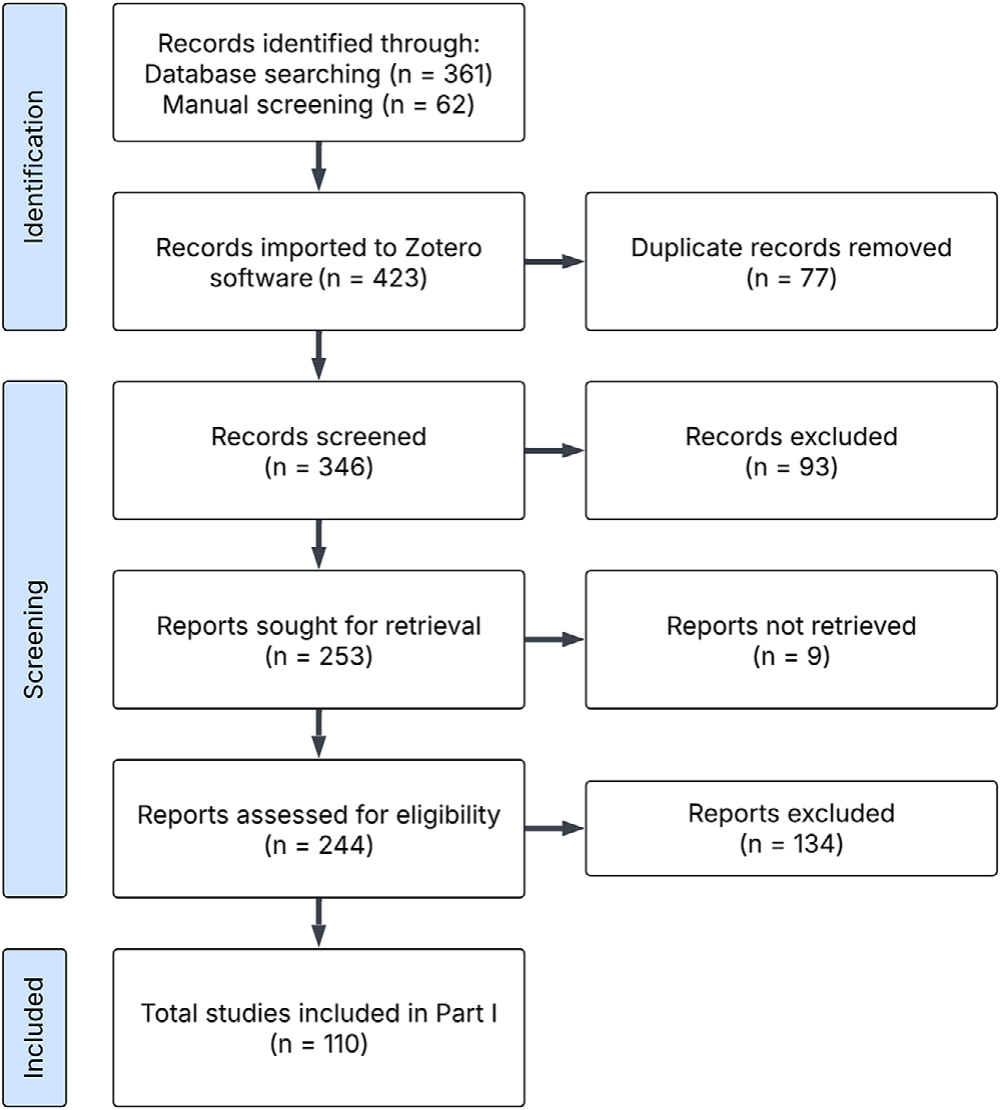
Flow diagram showing the process of identification, screening, eligibility assessment and inclusion of studies in the systematic review.

The final dataset included 110 eligible studies, published between 1991 and 2025, covering a wide range of species including dogs, cats, rabbits, birds, ruminants and reptiles. Flow diagram showing inclusion of studies is shown in Figure 1. For each included study, data were extracted using a standardized template capturing: bibliographic information, species, sample size, drug(s), dosage, method of IN delivery, outcomes and reported adverse reactions, as summarized in Tables 1, 2, 3, 4, 5, 6, 7.

**TABLE 1:** Summary of published studies on intranasal administration in dogs.

IN drug (s) [mg/kg]	Comments (results, advantages, disadvantages, method related notes)	Reference
**Sedation ± analgesia**		
Dex [0.02]	Both nostrils; INA stronger sedation; reduced bradycardia; practical non‐invasive alternative; ARNS	Micieli, Santangelo, Reynaud, et al. (2017); clinical; INA vs. IM; *n* = 10/group; SSC
Dex [0.02]	Both nostrils; INA lower plasma concentration; similar onset and sedation; less CV depression (possible CNS transport); struggling during IN; some drug swallowed	Santangelo et al. (2019); single‐blinded randomized crossover; INA vs. IM; *n* = 6; SSC; ♂ beagles
Med [0.04]	One nostril, considered less stressful; INA sedation comparable to IM; IND slower but cheaper; atomizer costly; priming increases drug use; IND: swallowing (5/6), nose licking (2/6); INA: ARNR	Jafarbeglou and Marjani (2019); single‐blinded randomized experimental; INA vs. IND vs. IM; *n* = 6/group
Dex [0.01] ± Ket [2] or Mid [0.3]	Both nostrils: authors suggested one‐nostril less stressful; Dex alone: adequate sedation; DexKet: deeper/faster sedation; DexMid: inconsistent with paradoxical excitement; nasal discomfort with all, worse with Ket or Mid; sneezing, swallowing, sialorrhea, licking and pawing at the nose; previous IN experience decreases tolerance and causes defensive reactions	Jafarbeglou, Marjani, Bakhshi‐Khanghah, et al. (2024); (1) double‐blinded randomized experimental; INA; *n* = 12/group; SSC (2) experimental (2 day after); INA; placebo; *n* = 36
Dex [0.005]	One nostril; INA comparable to IM in onset/sedation, effective at low dose, smooth/predictable sedation; sneezing and swallowing observed	López‐Ramis et al. (2022); single‐blinded randomized clinical; INA vs. IM; *n* = 8/group; SSC
Dex [0.005] ± methadone [0.3]	One nostril; adding methadone increased sedation and analgesia; HR decreased in INA/IM; 22% of dogs excluded (intolerance to atomizer); swallowing, sneezing, sialorrhea, retching, lip licking, reverse sneezing observed; high volume may increase runoff/swallowing	Bustamante et al. (2024); double‐blinded randomized clinical; INA; *n* = 10/group; SSC
Dex 125 µg/m^2^ (6 [range, 5–8] µg/kg)	For volumes > 0.5 mL split into both nostrils; INA safe and feasible for MRI premedication; maintained BP; reduced hypothermia; simple and well tolerated; bradycardia in two dogs; mild nasal scratching; unsuitable for aggressive/short‐muzzled dogs (requires snout holding); possible delivery failure	Lin et al. (2024); randomized clinical; INA vs. control, *n* = 13–15/group
Ket [2]	Both nostrils; Ket rapidly absorbed in Dex‐sedated dogs; full bioavailability; sedation lower than IV; HR increased in both (IV higher first 10 min); mild transient nasal irritation: sneezing, coughing, head shaking	Vlerick et al. (2020); randomized crossover; INA vs. IV, *n* = 7
Ket [10] + Med [0.05] or Dia [0.3]	Nostril NR; both decreased tear secretion and intraocular pressure (IOP); stronger effect with Med; ARNS; claims on speed, painlessness and safety not evaluated; only tear secretion and IOP evaluated; no SS used	Bulut and Yaygingul (2023); experimental; IND vs. IM, *n* = 7/group
**Analgesia**		
Fentanyl [4]	Both nostrils; INA may be an alternative to IV in emergencies; vocalization stopped within 2 min; respiratory rate decreased ∼30% (160 → 112); HR decreased ∼23% (120 → 92) at 7 min, PS improved (21 → 10) and dog laid down; ARNR	Micieli, Santangelo, Napoleone, et al. (2017); letter to the editor; case report, INA in one dog with acute pain
Tramadol [4]	Both nostrils; no differences in rescue analgesia, PS or physiologic variables; INA absorbed rapidly (Cmax 30–60 min, detectable up to 2 h); low plasma levels suggest direct CNS action; ARNR; both SS and PS used	Di Salvo et al. (2020); single‐blinded randomized clinical; INA vs. IV vs. IV methadone, *n* = 10/group
Buprenorphine [0.03]	One nostril; INA and oral transmucosal (OTM) achieved therapeutic plasma concentrations > 0.6 ng/mL for ∼5 and ∼4 h, respectively; IV maintained for > 9 h; IN and OTM showed no PK differences; both effective alternatives; sneezing in 2/5 dogs; nasal discharge (one dog, possible dose loss); PK study; no PS used	Enomoto et al. (2022); randomized crossover; INA vs. IV vs. OTM, *n* = 5; ♂ beagle‐mix dogs
**Reversal agents**		
Naloxone 4 mg/dog [0.17 ± 0.02 mg/kg]	One nostril; single‐dose INA rapidly absorbed (lag ∼2.3 min); INA dose ∼4 × IV; potential IV alternative in opioid emergencies; ARNR; only PK/PD, no reversal tested; no SS used	Wahler et al. (2019); randomized crossover; INA vs. IV 0.04 mg/kg: *n* = 6
Naloxone 4 mg/dog [0.13–0.19 mg/kg]	One nostril; both single‐dose INA and IM reversed IV fentanyl (0.3 mg/dog), SS decreased significantly; plasma levels lower with INA, but SS similar across groups; ARNR	Barr et al. (2023); single‐blinded crossover; INA vs. IM; *n* = 10
Naloxone 4 mg/dog	One nostril; single‐dose INA; odour detection ability unaffected from 2 to 48 h after reversal; no difference INA vs. IM; ARNR; only olfactory ability evaluated	Essler et al. (2019); randomized crossover; INA vs. IM; *n* = 10
Ati [0.3]	Both nostrils; successfully reversed sedation from IV xylazine 1.1 mg/kg; ARNR	Focken et al. (2024); experimental; INA; *n* = 6
Ati [0.2]	Both nostrils; reversed IM medetomidine (40 µg/kg) sedation; IM fastest, INA faster than IND but more costly; SS returned in all but HR did not return to baseline; IN easy for handler; feasible in emergencies or accidental exposures; ARNR: likely due to sedation	Jafarbeglou, Marjani, Oghbaei, et al. (2024); single‐blinded randomized clinical; INA vs. IND vs. IM, *n* = 10/group; SSC
**Seizure management**		
Levetiracetam [30]	Both nostrils; peak concenteration = 14.6 ± 5.4 µg/mL, time to peak = 2.3 ± 1.5 h; bioavailability = 70 ± 27%; therapeutic level (>5 µg/mL) reached in ∼0.3 h and maintained ∼6.5 h (vs. IV 9.8 h); sneezing observed in all; transient nasal congestion in one dog (1 day); only dogs that tolerated IN administration were included in the study	Wagner (2024); randomized crossover; INA vs. IV; *n* = 9
Dia [0.5]	Both nostrils; IND rapidly absorbed; plasma ≥ therapeutic (300 µg/L); useful when IV not available; onset ∼5 min, effect ≤ 15 min; IV caused immediate sedation/ataxia ≤ 15 min; IND caused milder sedation; PK study; ARNS	Platt et al. (2000); crossover; IND vs. IV; *n* = 6; Greyhounds
	Both nostrils; INA and IND achieved anticonvulsant concentrations (detectable at 2.5 min); INA easier and better tolerated than IND; blood levels comparable; half‐life shorter IN vs. IV; mild self‐limiting adverse effects (salivation, reverse sneezing, mild struggling); PK study	Musulin et al. (2011); crossover; IND vs. INA vs. IV, *n* = 6
Mid [0.2]	Both nostrils; IND gel superior to IND solution and rectal solution in plasma concentration and bioavailability; IV caused immediate sedation/ataxia (∼15 min duration); other routes onset ∼5 min, duration ≤ 15 min; both gel and solution effective for seizure treatment; ARNR; minimal nasal runoff observed with solution; PK study	Eagleson et al. (2012); crossover; solution 0.5% (IND vs. IV vs. rectal) vs. gel 5% (IND), *n* = 10; beagles
	Volumes > 1 mL split into both nostrils; INA terminated status epilepticus in 70% vs. 20% with rectal Dia 1 mg/kg; all dogs sedated with ataxia; INA significantly more effective for emergency seizure control; difficulties in 45% cases; sneezing in 78%; device use difficult in small nostrils; possible leakage	Charalambous et al. (2017); randomized clinical; INA (*n* = 20) vs. rectal Dia (*n* = 15); SSC
	Nostril NR; both routes quick, safe, effective for status epilepticus; INA superior when accounting for IV catheter placement time; sedation/ataxia in 88% (INA) vs. 79% (IV); brief sneezing 1/16 dogs (6%)	Charalambous et al. (2019); clinical; INA (*n* = 21) vs. IV (*n* = 23)
**Emesis induction**		
Apomorphine [0.06] (2× of other routes)	One nostril; IND provided rapid, easy, effective emesis; comparable efficacy to IV (88% vomited within 10 min IV vs. 72% IN); ARNR	Manley et al. (2024); randomized clinical; IND vs. IV vs. transconjunctival, *n* = 25/group
**Cannabidiol (CBD)**		
CBD 20 mg/dog	Both nostrils; INA vs. oral (100 mg) vs. rectal (100 mg); INA gave faster absorption (0.5 h) but lower levels; oral slower (3.5 h) but higher levels; rectal almost no absorption. sneezing (2 dogs); head shaking (1 dog)	Polidoro et al. (2022); randomized crossover; INA vs. rectal vs. oral, *n* = 6; beagles
**Clinically relevant animal model studies**		
**Benzodiazepines**		
Mid [1.5]	Both nostrils; IN routes resulted in higher CSF concentrations vs. plasma; INA produced higher CSF levels vs. IND; study under anaesthesia (limiting translation); sample size calculation (12 individuals/group) not implemented; 15 mg/mL, 3 × concentrated than commercial preparations; ARNS	Henry et al. (1998); crossover; metered dose INA vs. IND vs. IV; *n* = 6 (used twice)
Flurazepam, Mid, triazolam (doses not clearly stated)	Both nostrils; all absorbed across nasal mucosa without enhancers; INA gave rapid absorption and higher plasma peaks vs. oral; suggested useful for rapid hypnotic onset; ARNS	Lui et al. (1991); crossover; INA vs. Oral: *n* = 4; ♂ beagles
**Epinephrine**		
Phentolamine 5 mg/dog + Epi 28 mg/dog	Both nostrils; under pentobarbital anaesthesia; 3 min ventricular fibrillation then 7 min CPR with pneumatic chest compression; Epi given at 2 min into CPR; IN showed comparable effects on coronary perfusion pressure and survival as standard‐dose IV (0.015 mg/kg), suggesting a useful alternative when IV access is difficult; IN Phentolamine was given 1 min before Epi to enhance absorption; ARNS	Bleske et al. (1992); triple‐blinded randomized experimental; INC vs. IV; *n* = 10/group
Epi 2, 3, 4, 5, 10 or 20 mg/dog	One nostril; conscious dogs; IN absorption rapid, PK more variable especially at lower doses; IN caused blunted HR effects vs. IM and was well tolerated; HR remained elevated up to 90 min after IM; adverse effects: hypersalivation (most common), emesis, red nasal discharge, salivary pink staining	Dretchen et al. (2020a); crossover; 6 IND groups vs. 2 IM autoinjector groups (0.15 or 0.3 mg); total *n* = 20; *n* = 5–6/group
Epi 4 or 5 mg/dog	One nostril; Two studies: (1) 4 dogs, plasma and CSF PK (under anaesthesia) with rhinoscopy; (2) 16 dogs/group plasma PK and cardiovascular study in awake dogs; IN rapidly ↑ plasma levels vs. IM but no CSF reach; CV effects and HR similar to IM; rhinoscopy showed no significant mucosal damage (only mild erythema/vascularity); adverse effects (licking nose/muzzle, salivation, emesis/retching)	Dretchen et al. (2020b); experimental; IND vs. IM; *n* = 16/group
Epi 4 mg/dog ± nebulized histamine 5%	One nostril; dogs anesthetized; Epi given after histamine–induced nasal congestion; reduced congestion, showed faster absorption; earlier HR increase vs. placebo; congestion did not block absorption; ARNS	Tuttle et al. (2020); experimental; IND; *n* = 6/group
Epi 1 mg/dog	Probably one nostril (single‐dose INA); anesthetized dogs; during experimentally induced anaphylaxis with hypotension, IN showed increased absorption compared to normal state, likely due to increased vascular permeability; IN Epi remains effective during anaphylaxis; ARNS	Sparapani et al. (2023); crossover; INA, normal vs. anaphylactic dogs: *n* = 14

Abbreviations: Ati, atipamezole; ARNR, adverse reactions—none reported; ARNS, adverse reactions—not specified; CNS, central nervous system; CV, cardiovascular; Dex, dexmedetomidine; Dia, diazepam; Epi, epinephrine; HR, heart rate; IM, intramuscular; IN, intranasal; INA, intranasal atomization; INC, intranasal catheterization; IND, intranasal drops; Ket, ketamine; Med, medetomidine; Mid, midazolam; NR, not reported; PD, pharmacodynamic; PK, pharmacokinetic; PS, pain scale; SS, sedation score; SSC, sample size calculated; ♂, male only.

**TABLE 2:** Summary of published studies on intranasal administration in felines.

IN drug (s) [mg/kg]	Comments (results, advantages, disadvantages, method related notes)	Reference
**Domestic cats**		
Med [0.08]	Both nostrils; slower sedation onset vs. IM; otherwise comparable; sneezing in 1/7 cats; no SS used	Kaya and Yanmaz (2025); randomized experimental; INA vs. IM, *n* = 7/group; ♂; SSC
Dex [0.02]	Both nostrils; adequate sedation, reduced propofol need; IND had lower SS and required more anaesthetic; snorting or sneezing observed	Hommuang et al. (2022); clinical; IND vs. IM, *n* = 8/group; SSC
Dex [0.02] + morphine [0.2] or tramadol [1]	Both nostrils; both combos produced good sedation and comparable alfaxalone‐sparing; hypersalivation (not clearly distinguished from drug effects)	Hommuang et al. (2023); clinical; IND, *n* = 8/group; SSC
Dex [0.01]	Both nostrils; INA onset delayed vs. IM; no SS difference between groups; ARNS	Liu et al. (2025); single‐blinded randomized crossover; INA vs. IM, *n* = 8
Zolazepam‐tiletamine [10]	Both nostrils; onset and duration comparable; recovery faster with IND; sneezing observed; no SS used	Yanmaz, Doğan, Okumuş, et al. (2017); crossover; IND vs. IM, *n* = 8
	Both nostrils; recovery faster with IND; intraocular pressure (IOP) not affected by route; ARNS, only IOP evaluated, no SS used	Yanmaz et al. (2016); crossover; IND vs. IM, *n* = 8; ♂
Ket [14] + Mid [0.5]	Both nostrils; comparable onset, duration, recovery; faster sternal recumbency in recovery with IND; snorting/sneezing observed; no SS used	Marjani et al. (2015); experimental; IND vs. IM, *n* = 6/group
**Eurasian lynx (*Lynx lynx*)**		
Zolazepam–tiletamine [5]	Both nostrils; surgical anaesthesia (loss of withdrawal reflex) 29.4 min; sedation (righting reflex return) 104 min; allowed routine wound treatment; ARNR	Yanmaz, Doğan, Ersöz et al. (2017); letter to the editor; case report, IND

Abbreviations: ARNR, adverse reactions—none reported; ARNS, adverse reactions—not specified; Dex, dexmedetomidine; IM, intramuscular; IN, intranasal; INA, intranasal atomization; IND, intranasal drops; Ket, ketamine; Med, medetomidine; Mid, midazolam; SS, sedation score; SSC, sample size calculated; ♂, male only.

**TABLE 3:** Summary of published studies on intranasal administration in pigs.

IN drug (s) [mg/kg]	Comments (results, advantages, disadvantages, method related notes)	Reference
**Adult or weaned**		
Aza [2] or [4]	Nostril NR; INA 4 mg/kg sedation comparable to IM 2 mg/kg; ARNS	Svoboda et al. (2023); randomized experimental; INA vs. IM, *n* = 8/group; ♀ weaned piglets
Aza [3] + Mid [0.3] + Ket [7]	One nostril; IND effective as IM for chemical restraint; shorter onset and duration; minimal hemodynamic changes; one pig reacted to repeated IN; high volume (∼21 mL) caused swallowing; article supplemental video shows gargling sound	Rabelo et al. (2024); crossover; INC vs. IM; *n* = 16; ♂ immunocastrated landrace and large white pigs
Alf [1] or [2]	Both nostrils; increased SS; groups SS comparable; neither dose sufficient for handling or procedures; swallowing and drug loss at high volume (≤ 12.6 mL); 2 mg/kg hard to deliver	Hampton et al. (2021); single‐blinded randomized crossover; IND; *n* = 7; ♂ castrated Yucatan pigs; SSC
Mid [0.2] or [0.4]	One nostril; IND effective for sedation/anxiolysis; plasma concentration lower than IV in first 90 min; 0.4 mg/kg no added benefit; ARNS	Lacoste et al. (2000); (1) experimental; IND doses, *n* = 10/group; (2) crossover: IND vs. IV, *n* = 9; piglets (7.5 weeks)
Flunixin meglumine [2.2]	Probably one nostril; peak concentration = 2.7 µg/mL (IM 4.0 µg/mL); time to peak = 0.2 h; relative bioavailability = 88.5%; comparable to IM; minor inter‐animal variability in INA; ARNR	Wiloch et al. (2024); crossover; INA vs. IM; *n* = 6; ♀ Yorkshire pigs (8 weeks)
**Piglet castration anaesthesia**		
Aza [5] ± Alf [5] or Mid [0.2]	Nostril NR; INA not recommended; mild sedation with slower onset (> 10 min), shorter recovery, poor sedation depth, higher defence/vocalization scores; insufficient for major manipulations; difficult administration of large volumes: piglets showed massive defensive movements, discomfort and sneezing out drug; probably substances not fully absorbed in some cases; method may not be less stressful than IM	Breitenlechner et al. (2024); single‐blinded experimental; 3 INA vs. 9 IM groups, *n* = 10/group; piglets (4–7 days)
Aza [2] + S‐Ket [30]	Both nostrils; INA unsuitable for castration; recovery time comparable to IM; volume exceeded nasal capacity → incomplete absorption speculated	Becker et al. (2021); experimental; 1 INA, 3 IM, 2 control, *n* = 13/group; suckling piglets; SSC
Aza [1] + Ket [15] + climazolam [1.5]; Sarmazenil [0.2]	Both nostrils; INA faster recovery after reversal but higher reaction to surgical manipulations; most recumbent within 4–5 min; five piglets required extra dosing → delaying castration; sneezing in all piglets (↓drug availability); cold solution/room (< 18°C) likely reduced efficacy due to vasoconstriction; warming recommended	Axiak et al. (2007); single‐blinded randomized clinical; metered dose INA vs. IM, *n* = 20/group; suckling piglets
Flunixin meglumine [2.2]	One nostril, atomizer device connected to a bottle mount vaccinator; (1) IN flunixin ± lidocaine reduced post‐castration cortisol vs. controls; (2) no behavioural difference between groups, IN effective at reducing acute physiological stress post‐castration; ARNS	Lopez‐Soriano et al. (2023); single‐blinded clinical; INA (1) PK and cortisol; *n* = 24–25/group; toral *n* = 197; (2) behavioural; *n* = 59–60/group; Large White and Duroc piglets (9.0 ± 1.1 days)

Abbreviations: Alf, alfaxalone; Aza, azaperone; ARNR, adverse reactions—none reported; ARNS, adverse reactions—not specified; IM, intramuscular; IN, intranasal; INA, intranasal atomization; INC, intranasal catheterization; IND, intranasal drops; Ket, ketamine; NR, not reported; PK, pharmacokinetic; SS, sedation score; SSC, sample size calculated; ♂, male only; ♀, female only.

**TABLE 4:** Summary of published studies on intranasal administration in domestic and wild ruminants.

Species	IN drug (s) [mg/kg]	Comments (results, advantages, disadvantages, method related notes)	Reference
Cattle (calves)	Det [0.03]	Nostril NR; for calf disbudding under local anaesthesia; no calf reached score 4 on eyeball rotation; IND onset slower vs. IM, duration comparable; 1 calf not sedated; 2 coughed	Cinar et al. (2025); double‐blinded randomized experimental; IND vs. IM, *n* = 10; Holstein‐Friesian (15.6 ± 4.2 days); SSC
	Xyl [0.2]	One nostril; IN less consistent than IM/SC, longer onset, shorter duration; two individual sedation failures likely due to sneezing	Ede et al. (2019); crossover; IND vs. INA vs. IM vs. SC, *n* = 12; Holstein (6.2 ± 2.8 days)
Sheep	Det [0.01] or [0.03]	Nostril NR; INA reduced stress (lower cortisol); sedation comparable to IV but slower; dose‐dependent sedation; ARNS; criteria for onset not specified	Tahmasbi et al. (2023); single‐blinded (for SS only) experimental; INA vs. IV; *n* = 5/group; ♀ Lori sheep
	Buprenorphine 0.9 mg/animal	Probably both nostrils; rapid absorption; time to peak concentration ∼10 min; bioavailability 70%–89% depending on solvent; peak plasma level 37–48 ng/mL; short serum half‐life (10–23 min); could be clinically useful for pain management; ARNS	(Lindhardt et al. 2000); crossover; INA (2 solvents), *n* = 6; Icelandic sheep; sex NR
Southern mountain caribou (*Rangifer tarandus caribou*)	Med [0.1]	Nostril NR; 10 mg/mL; 10 × more concentrated than regular formulations; INA produced fast, predictable, reversible light sedation and muscle relaxation in net‐gunned, helicopter‐transported caribou; in field use since 2015; commercial laryngo‐tracheal atomizer used; training needed to avoid accidental staff exposure; ARNS	Mathieu et al. (2022); clinical; INA, *n* = 5
Manitoban elk (*Cervus canadensis manitobensis*)	Xyl [1.5–2.0] (estimated body weight)	One nostril; reduced handling stress in net‐gun captured elk; higher relaxation scores, decreased RR, cessation of struggling, relaxed limbs and lower cortisol, creatine kinase and gamma‐glutamyl transferase vs. controls; effect within 1 min; most stood/run after release (IV yohimbine administered before); ARNS	Cattet et al. (2004); single‐blinded experimental; IND vs. IND placebo; *n* = 20/group
White‐tailed deer (*Odocoileus virginianus*)	Naltrexone [1.5] + Ati [0.1] mg/kg	Naltrexone in one nostril, Ati in other; rapid effective reversal of medetomidine–carfentanil without resedation and renarcotization; IN vs. IM comparable in reversal time; slight delay in some IND endpoints; ARNS	Shury et al. (2010); crossover; INC vs. IM, *n* = 7; ♀

Abbreviations: ARNS, adverse reactions—not specified; Det, detomidine; IM, intramuscular; IN, intranasal; INA, intranasal atomization; INC, intranasal catheterization; IND, intranasal drops; NR, not reported; SS, sedation score; SSC, sample size calculated; Xyl, xylazine; ♀, female only.

**TABLE 5:** Summary of published studies on intranasal administration in rabbits, rats and mice.

IN drug(s) [mg/kg]	Comments (results, advantages, disadvantages, method related notes)	Reference
**Rabbits**		
Mid [1] + But [1.5] + Ket [5]	Both nostrils; IND: shorter onset and duration; no differences for SS, intraocular pressure, tear secretion and physiological variables; ARNR	Cinar and Yanmaz (2024); single‐blinded randomized crossover; IND vs. IM, *n* = 14, ♂ NZWR, SSC
But [0.5] + zolazepam‐tiletamine [15]	Both nostrils; IN onset/duration shorter vs. IM; SS, intraocular pressure and tear secretion comparable; ARNR	Cinar et al. (2024); single‐blinded experimental; IND vs. IM; *n* = 7/group; ♂ NZWR; SSC
Med [median doses 0.082, 0.163, 0.323]	INA Med 1 mg/mL: low dose: 0.3 mL one nostril; middle: 0.3 mL both; high: 0.3 mL twice both; produced dose‐dependent sedation and cardiorespiratory depression; loss of righting reflex: 1 rabbit (low dose), 7 (middle), 8 (high); volume‐dependent avoidance behaviour (controlled by physical restraint); sneezing in 1 rabbit	Wei, Chen, et al. (2023); single‐blinded randomized crossover; 3 INA groups vs. INA placebo, *N* = 8; ♀ JWR; SSC
Alfaxalone [median doses 3.1, 6.3, 9.4]	INA alfaxalone 4%: low: 0.15 mL both nares; mid: 0.3 mL both; high: 0.3 mL sequential (left, right, left); dose‐dependent sedation and respiratory depression (not clinically relevant); only high dose caused loss of righting reflex (all rabbits); volume‐dependent avoidance behaviour (controlled by physical restraint); recovery: 6/8 transient agitation/uncoordinated	Wei, Nakagawa, et al. (2023); single‐blinded randomized crossover; 3 INA groups vs. INA placebo, *n* = 8, ♀ JWR; SSC
But [0.4] + Mid [2] or Dex [0.1]	Probably both nares; ButDex: more effective sedation (higher SS); ButMid: longer sedation; both caused cardiopulmonary impairment; sneezing: 3 (ButDex), 1 (ButMid)	Okur et al. (2023); randomized crossover; IND, *n* = 8; ♂ NZWR; SSC
Ket [20] + Med [0.4] + But [0.2]; atipamezole [1–2]	Both nostrils; INA lighter sedation than IM; time to loss of righting reflex comparable; recovery faster with INA atipamezole; some brief struggling after INA; sneezing in 11/52	Degerfeld et al. (2023); clinical; INA vs. IM, *n* = 52/group; pet rabbits (various breeds); obese/cachectic excluded
Sufentanil [0.5] + Mid [2]	One nostril; IND and IM: moderate–deep sedation (∼13 min); allowed IV catheter placement and anaesthetic induction without resistance; ARNR	Freitag et al. (2022); single‐blinded randomized crossover; INC vs. IM, *n* = 11; ♀ NZWR
Dex [0.1] + Mid [2]	Both nostrils; IND faster onset and shorter duration vs. IM; sneezing in ∼50% with possible drug loss	Yanmaz et al. (2022); randomized crossover; IND vs. IM, *n* = 8; ♂ NZWR; SSC
Ket [10] + Med [0.2] or Dia [1]	Probably one nostril; KetDia: shorter loss/recovery times of reflexes; ARNS; no SS used	Bozkan et al. (2021); single‐blinded randomized experimental; INC, *n* = 6; NZWR
Med [0.2] + Ket [10] or S‐Ket [5]	Both nostrils; 71% adequately anesthetized for intubation after INC; some (8 MedKet, 11 MedS‐Ket) needed second dose; 1 MedKet and 3 MedS‐Ket required isoflurane; rapid onset, smooth recovery; sneezing, swallowing, head shaking; 2 rabbits (MedS‐Ket in dorsal recumbency) died with nasal haemorrhage (cause unclear: drug, preservatives, volume, IN catheter trauma or position)	Weiland et al. (2017); randomized experimental; INC in sternal (*n* = 42) vs. dorsal (*n* = 41); 2 centres; NZWR
Dex [0.1] + Mid [2] + But [0.4]	One nostril; IN catheter feasible/tolerated; provided strong restraint (∼45 min, suitable for painful procedures); some swallowing during dosing	Santangelo, Micieli, Mozzillo, et al. (2016); experimental; INC, *n* = 8; ♀ NZWR
	One nostril; plasma confirmed systemic absorption of all drugs; induced deep sedation and analgesia adequate for painful procedures; ARNS	Santangelo, Micieli, Marino, et al. (2016); experimental; INC, *n* = 5; ♀ NZWR
**Clinically relevant animal model studies**		
**Rabbits**		
Mid [0.5]	Both nostrils; rapid absorption (∼10 min), bioavailability 60%–70%; comparable to rectal route; female rabbits showed about 2 × higher CYP3A6 activity than males; ARNS	Zhuang et al. (2015); crossover; IND vs. IV vs. buccal vs. rectal; *n* = 6; NZWR
Buprenorphine 0.6 mg/rabbit	One nostril; bioavailability ∼46%–53% (solvent dependent); time to peak concentration 8–12 min; peak plasma level ∼27–28 ng/mL; ARNS	Lindhardt et al. (2001); crossover; IND (2 solvents), *n* = 6; NZWR; sex NR
Dia 3 mg/rabbit	Both nostrils; peak level reached after ∼5 min; very rapid pharmacodynamic response within 1.5–3.5 min; ARNS	Bechgaard et al. (1997); crossover; IND (various solvents); n NR; NZWR; sex NR
**Rabbits and rats**		
Dia [2]	Both nostril; PK study with brain tissue sampling; rabbits: rapid absorption into blood, then brain (time to peak concentration 5–10 min); results suggest most drug enters brain via blood circulation, not direct nose‐to‐brain route; ARNS	Kaur and Kim (2008); crossover; IND vs. IV; NZWR, SDR; various n; ♂
**Rats**		
Dex [0.01] + S‐Ket [0.2]	Dex left, Ket right nostril; 10‐day IN treatment reduced spinal inflammation markers (GFAP, TNF‐α) in diabetic neuropathy model; suggests potential for neuropathic pain management; ARNS	Sudo et al. (2025); experimental; IND vs. placebo; *n* = 6/group; ♂ WR
S‐Ket [0.5] or [1]	Both nostrils; under light halothane anaesthesia for IN administration; IN reduced pain behaviours in inflammatory and neuropathic pain models without affecting locomotion; stronger analgesia at 1 mg/kg; ARNS	Claudino et al. (2018); single‐blinded experimental; IND; *n* = 173; ♂ WR
Tramadol [5.2]	One nostril; PK study; rapid absorption and elimination; plasma and CSF levels high at 15 min; bioavailability 74% (conscious) and 67% (anesthetized); ARNS	Zhao et al. (2008); experimental; IND vs. IV vs. oral; n NR; ♂ SDR
Oxymorphone [1.1]	Nostril NR; PK study; rapid absorption; time to peak concentration ∼0.4 h; fast onset considered useful for analgesia; ARNS	Hussain and Aungst (1997); experimental; IND vs. IV; ♂ Lewis rats
**Mouse**		
(2R, 6R)‐hydroxynorketamine [10]	Both nostrils; produced significant analgesia in acute pain tests; showed mild anxiolytic effect without sedation or cardiovascular changes, suggesting safe pain relief profile; ARNS	Goswami et al. (2023); single‐blinded experimental; IND; *n* = 10/group; ♂ C57BL/6J mice
Ket [1] or [5] or [20]	Nostril NR; increased pain threshold in thermal and formalin tests; no major cardiovascular or EEG changes; mild sedative effect at higher doses, with slightly reduced locomotion; ARNS	Goswami et al. (2021); single‐blinded experimental; IND vs. control; *n* = 7/groups; ♀ C57BL/6J mice

Abbreviations: ARNR, adverse reactions—none reported; ARNS, adverse reactions—not specified; But, butorphanol; Dex, dexmedetomidine; Dia, diazepam; IM, intramuscular; IN, intranasal; INA, intranasal atomization; INC, intranasal catheterization; IND, intranasal drops; JWR, Japanese white rabbit; Ket, ketamine; Med, medetomidine; Mid, midazolam; NZWR, New Zealand white rabbit; NR, not reported; PK, pharmacokinetic; SDR: Sprague–Dawley rats; SS, sedation score; SSC, sample size calculated; WR; Wistar rats; ♂, male only; ♀, female only.

**TABLE 6:** Summary of published studies on intranasal administration in birds.

Species	IN drug (s) [mg/kg]	Comments (results, advantages, disadvantages, method related notes)	Reference
**Passeriformes**			
Domestic canary (*Serinus canaria forma domestica*)	Dia or Mid or Xyl or Det; Flu or Yoh; [range used]	Both nares; BZD sedation within 1–2 min, dorsal recumbency (Dia longer); α2‐agonists: heavy sedation, sternal recumbency only; higher doses prolonged sedation, no dorsal recumbency; α2‐agonists longer duration (Det longest), Mid shortest; Flu shortened BZD sedation; Yoh antagonized α2‐agonists; ARNR; no SS used; side test contrast radiography confirmed drug stayed in nasal cavity	Vesal and Zare (2006); crossover studies with dose detection; IND groups, total *n* = 26; *n* = 5/group
Zebra finch (*Taeniopygia guttata*)	Xyl [27 ± 2.8] or Dia [13.0 ± 1.0] or Mid [13.0 ± 1.0]	Naris NR; BZD adequate sedation for diagnostic/minor procedures; Mid fastest onset; Xyl longest sedation, no dorsal recumbency; myoclonic convulsions reported; ARNR; no SS used	Bigham‐Sadegh and Zamani‐Moghadam (2013); single‐blinded crossover; 3 IND groups, *n* = 16
Red‐billed chough (*Pyrrhocorax pyrrhocorax*)	Xyl [8] or Dia [8] or Mid [8] ±/or Ket [30]	Both nares; all groups sedated; Xyl ± Ket: longest onset and poor recovery; Xyl longest dorsal recumbency; BZD shortest sedation; ARNS; no SS used	Raisi et al. (2016); crossover; 7 IND groups; *n* = 7
		Naris NR; HR was lowest in Xyl or XylKet; ARNS; only electrocardiography evaluated; no SS used	Taati and Raisi (2017); crossover; 7 IND groups; *n* = 10
**Psittaciformes**			
Ring‐necked parakeet (*Psittacula krameri*)	(Xyl or Mid ± Ket) or Det; Ati, Yoh, Flu; [range of doses used]	Single drug both nares; combos split (sedative right, Ket left); Mid protocols preferred, reliable sedation, rapid onset, acceptable depth, faster recovery, safe; MidKet deep analgesia; α2‐agonists prolonged, less practical sedation, residual effects, sluggishness and insufficient depth for dorsal recumbency; reversal agents shortened sedation; ARNS; Flu after MidKet: transient excitement/self‐injury; tremors; no SS used; contrast radiography confirmed IN drug retention, oral Mid ineffective	Vesal and Eskandari (2006); Crossover studies with dose detection; IND groups, total *n* = 17; *n* = 6/group
Budgerigar (*Melopsittacus undulatus*)	Xyl [26] or Dia [12] ±/or chitosan	Both nares; Xyl rapid sedation, sternal recumbency, no dorsal (even at 50 mg/kg); Dia rapid, heavy sedation, dorsal recumbency; chitosan prolonged sedation/dorsal recumbency with both; ARNS; no SS used	Al‐Shebani (2011); experimental studies: (1) dose (4 IND; *n* = 6/group); (2) chitosan additive (4 IND; *n* = 6/group)
	Xyl [25.6 ± 2.2] or Dia [13.6 ± 1.1] or Mid [13.2 ± 1.3]	Both nares; Mid fastest onset, but shorter dorsal recumbency vs. Dia; Xyl longest sedation, but no dorsal recumbency; ARNR; no SS used.	Bigham‐Sadegh (2013); single‐blinded crossover; 3 IND groups: *n* = 15
	Mid [5] + Ket [15] or S+Ket [15]	One naris; both IN and IM combos effective for short, non‐invasive procedures; IN: shorter decubitus and anaesthesia vs. IM; sneezing in ∼48% after IN; insulin syringe caused small nostril lesions with mild, self‐limiting bleeding in seven birds	Trevisan et al. (2016); Portuguese language article, crossover; IND vs. IM, *n* = 8
Hispaniolan (*Amazona ventralis*) amazon parrot	Mid [2]; Flu [0.05]	Probably both nares; Mid: fast, reliable, moderate–deep sedation; Flu enabled rapid, full recovery; ARNR	Mans et al. (2012); single‐blinded (for SS) crossover; IND vs. placebo: *n* = 9
Blue‐fronted (*Amazona aestiva*) amazon	Ket [15] + Mid [1]	Ket right, Mid left naris; IN: shorter onset; IM: longer sedation and recovery; sedation quality/duration similar between routes; sneezing in one IN bird, likely ↓ absorption	Bitencourt et al. (2013); Portuguese language article, single‐blinded experimental; IND vs. IM; *n* = 7/group; exact species distribution NR
Vinaceous‐breasted (*Amazona vinacea*) amazon			
Blue‐fronted (*Amazona aestiva*) amazon	Mid [2]	Both nares; effective sedation, short onset, fast recovery in most; three blue‐fronted not sedated; sneezing in two blue‐fronted	Schaffer et al. (2017); experimental; INC; *n* = 10/species; wild‐caught
Orange‐winged (*Amazona amazonica*) amazon			
Blue‐and yellow macaw (*Ara araruana*)		Both nares; vocalization reduced/absent within ∼61 seconds; onset ∼2 min; 10/13 (76.9%) spontaneously assumed lateral recumbency; total sedation time ∼20.0 min; SS in 10 birds 3, 1 bird 2, 2 birds 1; 9/13 side effects (sneezing 5; airway noise 2; trembling 2), all resolved after recovery	Schaffer et al. (2016); letter to the editor: INC, *n* = 13
Cockatiel (*Nymphicus hollandicus*)	Mid [3] ± But [3]; Flu [0.05]	Both nares; Mid and MidBut: induced sedation; MidBut deeper during rest/restraint; respiratory rate ↓, no effect on HR/temp; SS ↓after Flu but was higher than baseline values; ARNR	Doss et al. (2018); double‐blinded randomized crossover; 2 IND groups vs. IND placebo, *n* = 9
	Alf [15]	One naris; IND did not produce clinically relevant sedation; sneezing shortly after dosing; possible drug expulsion	Jones et al. (2025); single‐blinded randomized crossover; IND vs. IM, *n* = 8; ♂
Quaker parrot (*Myiopsitta monachus*)	Mid [2] + But [2] + (IM Alf)	Naris NR; IND MidBut + IM Alf: safe and effective; ARNS; IND without IM Alf not tested	Conner et al. (2022); single‐blinded randomized crossover; IND combo, *n* = 10
**Anseriformes**			
Mallard duck (*Anas platyrhynchos*)	Alone or combos: medetomidine, Xyl, Ket, Mid, But, fentanyl, sufentanil, methohexital, Alf‐alphadolone, propofol; Ati, Flu	IND: no effect or short sedation/anaesthesia vs. IM/IV; swallow or cough after IN	Machin and Caulkett (1998); crossover; IND, IM, IV; dose detection
Surf scoter (*Melanitta perspicillata*)	Mid [4.6–5.9]	Naris NR; field use; IN after capture for transmitter implant; improved post‐surgical survival; ARNS	Net et al. (2019); clinical; INC vs. placebo, *n* = 26/group; ♀
**Galliformes**			
Japanese quail (*Coturnix coturnix japonica*)	Mid [6] + Ket [100]	Naris NR; onset: IO > O > IM > IND; duration: IND and O > IO > IM; ARNS; anaesthesia subjectively evaluated by needle prick	Yayla et al. (2018); Experimental; IND vs. IM vs. Oral (O) vs. Intraosseous (IO), *n* = 8/group; ♂
Domestic Chicken (*Gallus gallus domesticus*)	Dex [0.08] ± But [2]	Both nares; Dex effective/short duration; DexBut slower absorption; But alone: low maximum plasma concentration, poor analgesia; ARNS	Sha et al. (2022); experimental; INC; *n* = 6/group; (2) single‐blinded experimental; INC vs. placebo, *n* = 6/group; ♂ Ross broilers
	Ket [30] + Mid [2]	One naris; IND faster recovery; less consistent sedation; less agitation/vocalization; sneezing likely but not well reported; one bird failed lowest SS; only 5/12 reached max SS	Adão et al. (2024); Single‐blinded crossover; IND vs. IM, *n* = 12; Sex not reported
**Columbiformes**			
Pigeon (*Columba livia domestica*)	Xyl [29.4 ± 1.9] or Dia [6.4 ± 0.9] or Mid [6.5 ± 1.0]	Naris NR; Mid: fastest onset; BZD: adequate sedation; Xyl longest sedation but no dorsal recumbency; ARNS; no SS used	Zamani‐Moghadam et al. (2009); crossover; IND, *n* = 15
	Mid [5] ± Dex [0.08]; Ati [0.25]	Probably both nares; Mid: minimal side effects, inadequate immobilization, excitation on recovery; MidDex: effective immobilization (20–30 min), tolerated posture changes; Ati antagonized both sedation and side effects but full recovery not achieved within 10 min; ARNS	Hornak et al. (2015); experimental; IND, *n* = 6/group; unsexed
	Acepromazine [0.5] or [1] or Dia [5]	Probably both nares; Dia: fastest onset; Acepromazine 1: longest duration; Acepromazine 0.5: shortest duration; ARNS; sedation depth subjectively evaluated	Vajdi and Alian‐Samakkhah (2023); single‐blinded experimental; IND, *n* = 10/group
	Mid [4] + But [2]	Both nares; only intraocular pressure assessed; IND and IM were comparable, intraosseous showed significant decrease; ARNR	(Yener and Hurma 2025); experimental; IND vs. IM vs. Intraosseous; *n* = 8/group
**Raptors**			
Common Buzzard (*Buteo buteo*)	Mid [2]; Flu [0.05]	Both nares; Mid: short onset, mild–moderate sedation (not deep); Flu: rapid recovery; side effects: sneezing‐like (3/10), wing fluttering (2/10)	Altundag et al. (2021); experimental; INC vs. placebo, *n* = 10/group
Great horned owls (*Bubo virginianus*)		Both nares; no significant difference in sedation/muscle relaxation between IN and IM; at 15 min after Flu, scores seem same as prior timepoints; all owls recovered and returned enclosures < 2 h; onset and jaw tone criteria unclear; ARNR	Hawkins et al. (2025); Single‐blinded randomized crossover; IND vs. IM, *n* = 6
**Others**			
Ostrich (*Struthio camelus*)	Dia or Mid [0.2–1.6] or Xyl [0.5–4]	Both nares; mild sedation with low doses (Dia 0.2–0.4, Mid 0.2, Xyl 1); moderate with Dia 0.8, Mid 0.4, Xyl 2; deep (recumbency) with Mid 0.8, Xyl 2; Mid: shorter onset and longer duration vs. Dia/Xyl; preferred dose: 0.4 for standing, 0.8 for recumbency; BZD ≥ 1.6 not tolerated (large volume → head shaking, drip out); Xyl 0.5 ineffective	Araghi et al. (2016); randomized experimental; 12 IND groups, *n* = 6/group; juvenile ostriches (4–5 months)

Abbreviations: Alf, alfaxalone; Ati, atipamezole; ARNR, adverse reactions—none reported; ARNS, adverse reactions—not specified; BZD, benzodiazepine; But, butorphanol; Det, detomidine; Dex, dexmedetomidine; Dia, diazepam; Flu, flumazenil; IM, intramuscular; IN, intanasal; INC, intranasal catheterization; IND, intranasal drops; Ket, ketamine; Mid, midazolam; NR, not reported; SS, sedation score; Xyl, xylazine; Yoh, yohimbine; ♂, male only; ♀, female only.

**TABLE 7:** Summary of published studies on intranasal administration in reptiles.

Species	IN drug(s) [mg/kg]	Comments (results, advantages, disadvantages, method related notes)	Reference
Green iguana (*Iguana iguana*)	Mid [2] or [3] or [5]	Both nostrils; IN 2–3: no sedation; IN 5: mild, inconsistent (20–90 min); IM: mild–deep; adverse: sneezing, nasal discharge, head movements, escape; drug loss suspected	Sarri et al. (2025); randomized crossover; 3 INC vs. IM vs. IM placebo; *n* = 15; sex not reported
Red‐eared slider (*Trachemys scripta elegans*)	(1) Dex [0.2] + Ket [10]; Ati [2]; (2) Alf [5]	Ket left, Dex right; Ati and Alf both nares; KetDex: safe sedation; head‐in vs. head‐out: no diff; Ati: rapid reversal < 10 min, HR ↑; Alf: HR ↓, no reflex loss; ARNS	Cermakova et al. (2018); crossover; IND; head‐in (*n* = 14) vs. head‐out (*n* = 16) vs. control (*n* = 5); ♀
Yellow‐bellied slider (*T s scripta*)	Dex [0.2] + Ket [10]; Ati [2]	Ket left, Dex right, A both nares; KetDex: moderate–deep, flaccid limbs/head, procedures feasible; Ati: effective reversal; high k and Dex interindividual plasma variability; large volume → possible loss (oropharynx) → reduced SS	Schnellbacher et al. (2012); experimental; 1 IND group; *n* = 8
	Mid [1–2]	Same article conducted another trial with Mid in six turtles; poor sedation; 4/6 oral froth (irritation); trial stopped	
Red‐footed tortoise (*Chelonoidis carbonaria*)	Dex [0.05] or [0.15] or Mid [0.5] or [1.5]	One or both nares (depending on volume); both ineffective; large volumes difficult to deliver; frequent drug loss (sneezing, nasal blowing, head movements); only small concentrated vol may be effective	Emery et al. (2014); single‐blinded crossover; 4 IND drugs vs. IND placebo; *n* = 6/species
Indian star tortoise (*Geochelone platynota* ^a^)			
Russian tortoise (*Testudo horsfieldii*)	Dex [0.2] + Ket [10]; Ati [0.5]	Ket left, Dex right, Ati both nares; tortoises: activity/reflexes intact, HR ↓; terrapins: safe sedation, loss of head withdrawal and glottal reflex (ET intubation feasible), HR ↓; Ati: rapid reversal, HR ↑; head‐in vs. head‐out: no difference; ARNS	Knotek and Cermakova (2014); IND; conference abstract; experimental; *n* = unknown
Hermann's tortoise (*Testudo hermanni*)			
Red‐eared terrapin (*T s elegans*)			

Abbreviations: Alf, alfaxalone; Ati, atipamezole; ARNS, adverse reactions—not specified; Dex, dexmedetomidine; HR, heart rate; IM, intramuscular; IN, intranasal; INC, intranasal catheterization; IND, intranasal drops; Ket, ketamine; Mid, midazolam; SS, sedation score; ♀, female only.

^a^ Likely species was *Geochelone elegans*; use of *G. platynota* in the article may represent misidentification, since *G. platynota* refers to the critically endangered Burmese star tortoise.

To ensure consistent classification, studies were categorized using predefined criteria rather than studies’ self‐labelling. Trials were considered clinical if conducted in routine practice; otherwise, they were deemed experimental. Experimental studies were defined as crossover if a washout period were explicitly reported. Studies were classified as randomized if the method used to generate the allocation sequence was clearly described. They were considered blinded if blinding procedures were explicitly stated and the individuals aware of group allocation at different stages were clearly identified: single‐blinded (outcome evaluators), double‐blinded (evaluators and treatment providers) or triple‐blinded (evaluators, providers and statisticians). Sample size was considered appropriately calculated only when sufficient methodological detail and valid assumptions were reported.

Formal methodological quality assessment and risk of bias analysis were not performed, as these were beyond the scope of the current review. Consequently, included studies may carry variable methodological and reporting biases. Readers are therefore encouraged to critically appraise individual studies before extrapolating their findings to research or clinical practice.

### 1.2 Part I: IN Administration in Animal Species

#### 1.2.1 Dogs

IN administration of sedatives, analgesics and emergency drugs has been extensively studied in dogs (Table 1). Among these, dexmedetomidine and medetomidine are the most frequently studied agents. Their IN administration generally produces sedation comparable to IM route, while being easier to perform and, in some studies, associated with reduced bradycardia and cardiovascular depression (Jafarbeglou and Marjani 2019; Lin et al. 2024; López‐Ramis et al. 2022; Micieli, Santangelo, Reynaud, et al. 2017; Santangelo et al. 2019). Addition of ketamine or methadone deepens sedation; whereas co‐administration with midazolam has yielded variable results, including occasional paradoxical excitement (Bustamante et al. 2024; Jafarbeglou, Marjani, Bakhshi‐Khanghah, et al. 2024). Opioids such as buprenorphine and tramadol delivered IN reach therapeutically useful levels and provide clinical analgesia despite lower plasma concentrations versus IV (Di Salvo et al. 2020; Enomoto et al. 2022).

IN delivery is also valuable in emergencies: commercial IN naloxone is rapidly absorbed and effective for opioid reversal (Barr et al. 2023; Essler et al. 2019; Wahler et al. 2019), while IN atipamezole reliably antagonizes α2 adrenergic agonists’ sedation (Focken et al. 2024; Jafarbeglou, Marjani, Oghbaei, et al. 2024). IN apomorphine provides a practical emetic option when IV is not feasible (Manley et al. 2024). For seizure management, IN diazepam and midazolam rapidly achieve anticonvulsant concentrations, offering effective and often superior seizure control compared with rectal administration (Charalambous et al. 2019, Charalambous et al. 2017; Eagleson et al. 2012; Musulin et al. 2011; Platt et al. 2000). Earlier animal‐model research demonstrated rapid nasal absorption of benzodiazepines with high cerebrospinal fluid exposure (Henry et al. 1998; Lui et al. 1991). Recent investigations have extended IN applications to cannabidiol and levetiracetam (Polidoro et al. 2022; Wagner 2024). Finally, several animal‐model studies show IN epinephrine achieves rapid absorption comparable to IM/IV during cardiopulmonary resuscitation or anaphylaxis (Bleske et al. 1992; Dretchen et al. 2020a, 2020b; Sparapani et al. 2023; Tuttle et al. 2020).

#### 1.2.2 Felines

In cats (Table 2), medetomidine (Kaya and Yanmaz 2025), dexmedetomidine alone (Hommuang et al. 2022; Liu et al. 2025) or with opioids (Hommuang et al. 2023) and dissociative‐benzodiazepine combinations (Marjani et al. 2015; Yanmaz, Doğan, Ersöz, et al. 2017, Yanmaz, Doğan, Okumuş, et al. 2017, Yanmaz et al. 2016) were well tolerated and produced effective sedation. Sedation onset was slower than IM but of comparable quality.

#### 1.2.3 Pigs

In adult or weaned pigs (Table 3), azaperone alone or with midazolam or ketamine provided sedation broadly comparable to IM, though high volumes often led to swallowing (Rabelo et al. 2024; Svoboda et al. 2023). While alfaxalone produced only mild and insufficient sedation (Hampton et al. 2021), midazolam reported to be effective for sedation/anxiolysis (Lacoste et al. 2000). On other hand, for piglet castration anaesthesia, azaperone‐based protocols were generally unsatisfactory (Axiak et al. 2007; Becker et al. 2021; Breitenlechner et al. 2024).

#### 1.2.4 Ruminants

Data for domestic and wildlife ruminants are shown in Table 4. In neonatal calves and adult sheep detomidine provided clinically relevant sedation, whereas xylazine in calves was less consistent and often unreliable (Cinar et al. 2025; Ede et al. 2019; Tahmasbi et al. 2023). Field studies investigating IN xylazine and medetomidine in Cervidae have demonstrated rapid, predictable sedation, with improved stress markers compared to controls following net gun capture (Cattet et al. 2004; Mathieu et al. 2022). In addition, IN naltrexone‐atipamezole has been shown to effectively reverse medetomidine‐carfentanil immobilization (Shury et al. 2010).

#### 1.2.5 Small Mammals

In rabbits (Table 5), dexmedetomidine (0.1 mg/kg) or its equipotent doses of medetomidine (0.2 mg/kg), ketamine (10–20 mg/kg), butorphanol (0.2–0.5 mg/kg) and midazolam (2 mg/kg) are the most frequent used drugs in combination (Cinar et al. 2024; Cinar and Yanmaz 2024; Degerfeld et al. 2023; Okur et al. 2023; Santangelo, Micieli, Mozzillo, et al. 2016; Santangelo, Micieli, Marino, et al. 2016; Yanmaz et al. 2022). While medetomidine alone produces dose‐dependent sedation (Wei, Chen, et al. 2023), alfaxalone was clinically ineffective (Wei, Nakagawa, et al. 2023). An early report, available only in abstract form, noted that IN fentanyl/droperidol caused severe adverse effects and high mortality, and its use is not recommended (Robertson and Eberhart 1994). IN midazolam is also described in exotic species such as hedgehogs and sugar gliders (Doss and De Miguel Garcia 2022). For rodents, although most studies derive from pharmaceutical models, selected reports with clinical relevance (as identified by the author) are summarized in Table 5.

#### 1.2.6 Birds

IN sedation in birds shows highly variable efficacy across species, drugs and doses (Table 6). Across a wide range of taxa, midazolam generally provides short‐acting, reversible sedation suitable for handling or minor procedures. In contrast, α2 adrenergic agonists are less predictable and often impractical due to slow onset and residual sedative effects (Altundag et al. 2021; Araghi et al. 2016; Net et al. 2019; Vesal and Eskandari 2006; Vesal and Zare 2006). Combining midazolam or diazepam with opioids or ketamine can enhance sedation depth and reliability (Doss et al. 2018; Raisi et al. 2016). Reversal agents are also effective: flumazenil rapidly antagonizes benzodiazepines (Hawkins et al. 2025; Mans et al. 2012), and yohimbine or atipamezole can reverse α2 adrenergic agonists (Hornak et al. 2015; Vesal and Eskandari 2006).

Despite abundant evidence, many avian reports remain poorly documented or appear only as personal observations in guidelines. For instance, one author reported that, combining midazolam (2 mg/kg) with butorphanol (0.5–2.0 mg/kg, IM or IN) deepens sedation in Galliformes but increases cardiopulmonary depression (Heard 2025). At a Raptor Centre, IN midazolam in bald eagles (*Haliaeetus leucocephalus*) and other raptors produced inconsistent effects compared with IM (Willette et al. 2025). A personal observation reported mild‐to‐moderate sedation with IN midazolam in little blue and Fiordland penguins (Bodley and Schmitt 2025), and its use has been reported in a yellow‐collared macaw (*Primolius auricollis*, Coutant et al. 2018).

#### 1.2.7 Reptiles

In reptiles (Table 7), IN midazolam generally failed to produce adequate sedation, often causing only mild effects or adverse reactions such as oral discharge (Emery et al. 2014; Sarri et al. 2025; Schnellbacher et al. 2012). Dexmedetomidine alone was ineffective (Emery et al. 2014), and ketamine alone has not been evaluated. However, dexmedetomidine and ketamine combinations produced clinically useful sedation in terrapins, reversible with atipamezole, whereas no significant effect was observed in tortoises (Cermakova et al. 2018; Knotek and Cermakova 2014; Schnellbacher et al. 2012).

### 1.3 Part II: IN Delivery Techniques and Practical Challenges

**FIGURE 2:**
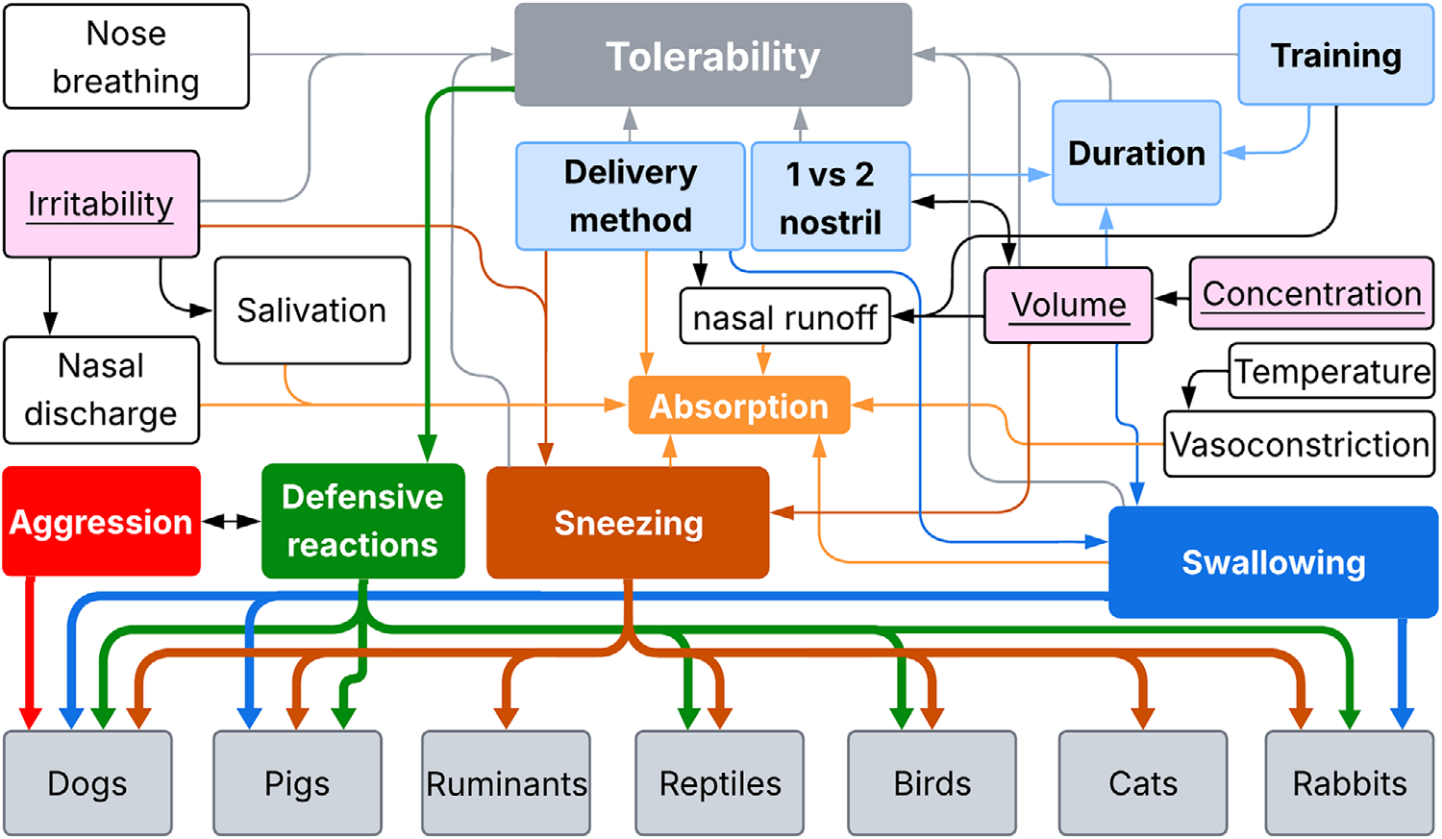
Conceptual summary illustrating factors that influence intranasal drug delivery, their interrelationships and their potential impact on absorption and tolerability. Factors related to the administration technique are shown in light blue rectangles with bold black font, those related to the drug or formulation are shown in pink rectangles with underlined black font, and other factors, including physiological and environmental variables, are shown in white rectangles with black font. The direction of the arrows indicates the influence of the starting rectangle on the ending rectangle (positive or negative effect). Major factors and commonly reported adverse reactions are emphasized with full coloured rectangles and bold white font. Representative adverse events and the species in which they were reported are noted at the bottom of the figure.

The main factors influencing IN drug delivery, associated challenges and adverse events are summarized in Figure 2, which illustrates their interrelationships and potential impact on absorption and tolerability. The following sections discuss these factors in detail.

#### 1.3.1 Interspecies Anatomical and Physiological Differences

A frequently overlooked factor influencing IN administration over species is the anatomical evolution of the nasal cavity. Mammals generally possess more complex nasal and concha structures than birds (Xi et al. 2023), whereas many reptiles have simplified anatomy or lack conchae entirely (Martinez et al. 2024). Greater concha complexity increases absorptive surface area (Gizurarson 2012, 1990), which may explain the higher efficacy of IN delivery in mammals and birds compared with reptiles.

On the other hand, the olfactory epithelium has been proposed as a potential route for direct brain delivery in animals (Bustamante et al. 2024; Micieli, Santangelo, Reynaud, et al. 2017; Wei, Nakagawa, et al. 2023). However, in macrosmatic species with highly developed concha systems such as dogs, rabbits and rats (Xi et al. 2023), access to the olfactory region located deep within the ethmoidal conchae is limited (Gizurarson 2012, 1990; Wei et al. 2022). In rabbits and rats, drugs appear to reach the brain primarily through systemic absorption rather than direct nasal‐to‐brain transport (Kaur and Kim 2008), with little or no deposition on the nasal conchae (Wei et al. 2022). Therefore, while complex concha anatomy may enhance systemic absorption, it can also hinder direct nose‐to‐brain delivery compared with primates (Gizurarson 2012, 1990).

Species‐specific respiratory physiology is another critical consideration. In contrast to humans and dogs which can alternate between nasal and oral respiration; mammalian neonates and adults in other species such as cats, rabbits, rodents, equids, suids, camelids, ruminants, chelonians and birds exhibit anatomical and physiological adaptations that render them obligate or near‐obligate (preferential) nasal breathers (Fröhlich 2024; Kerr and Teixeira‐Neto 2024; Mazan 2022; Trabalon and Schaal 2012; West et al. 2025). In these animals, IN administration, particularly when large volumes are delivered bilaterally, may cause significant distress and potentially compromise airway patency (Santangelo, Micieli, Marino, et al. 2016). In one rabbit study, two animals died after IN dosing in dorsal recumbency; although the exact cause was undetermined, the potential role of airway obstruction cannot be excluded (Weiland et al. 2017).

#### 1.3.2 Delivery Method

Three main techniques are reported for IN administration (Table 8): (1) the IN drops (IND) technique (2) IN atomization (INA) and (3) IN catheterization (INC).

**TABLE 8:** Comparison of three intranasal administration methods: Intranasal atomization (INA), intranasal drops (IND) and intranasal catheterization (INC).

Method	Principle	Advantages	Limitations	Typical species
**IND**	Solution instilled via syringe or pipette directly into nostril	Simple, cheap, widely available; no device‐specific constraints; feasible across most species	Slower onset, lower absorption vs. INA in some studies; higher risk of runoff and swallowing; larger volumes poorly tolerated; may require more prolonged restraint	Can be used in both large and small sized species
**INA**	Produces fine mist for wider mucosal coverage	Increased surface area and spread; potentially faster absorption/onset; reduced caudal runoff; often considered more comfortable	Requires proper nozzle fit and orientation; limited feasibility in small animals (cats, rabbits, brachycephalic dogs); higher cost; misuse can mimic IND (no atomization); noise may stress animals	Preferable in larger species; less or no effective in small species
**INC**	Catheter inserted into nasal cavity (ventral meatus)	May reduce rostral runoff; potential for more targeted deposition; may allow larger volumes vs. IND	More invasive, uncomfortable, can trigger sneeze reflex; requires more restraint and skill; risk of trauma, bleeding or drug swallowing; rare reports of mortality in studies	Mainly used rabbits, cats, birds; research use rather than routine practice

#### 1.3.3 Intranasal Drops (IND)

This method uses a pipette or needleless syringe to deliver drops directly into the nostrils (Musulin et al. 2011; Vesal and Zare 2006). It is simple, inexpensive and feasible in small species. However, IND is often associated with slower onset and weaker efficacy, probably due to higher caudal runoff (Jafarbeglou, Marjani, Oghbaei, et al. 2024; Jafarbeglou and Marjani 2019).

#### 1.3.4 Intranasal Atomization (INA)

INA uses mucosal atomizers, also known as sprays, to create a fine mist (30–100 µm). The aerosol increases mucosal surface contact, improves distribution and promotes faster absorption while reducing caudal runoff compared with IND (Musulin et al. 2011; Wagner 2024). Some studies report faster onset or higher cerebrospinal fluid to plasma concentration ratios with INA (Henry et al. 1998; Jafarbeglou and Marjani 2019), whereas others find no clear advantage over IND (Ede et al. 2019; Musulin et al. 2011). Similarly, although atomizers are sometimes claimed to improve animal comfort (Musulin et al. 2011), other studies have found no significant difference in ease of administration, as assessed by animal resistance scales (Jafarbeglou and Marjani 2019; López‐Ramis et al. 2022). A potential limitation of INA is the noise from spraying (Jafarbeglou et al. 2018), which may cause stress in certain animals.

#### 1.3.5 MAD Nasal Atomizer: Advantages and Disadvantages

**FIGURE 3:**
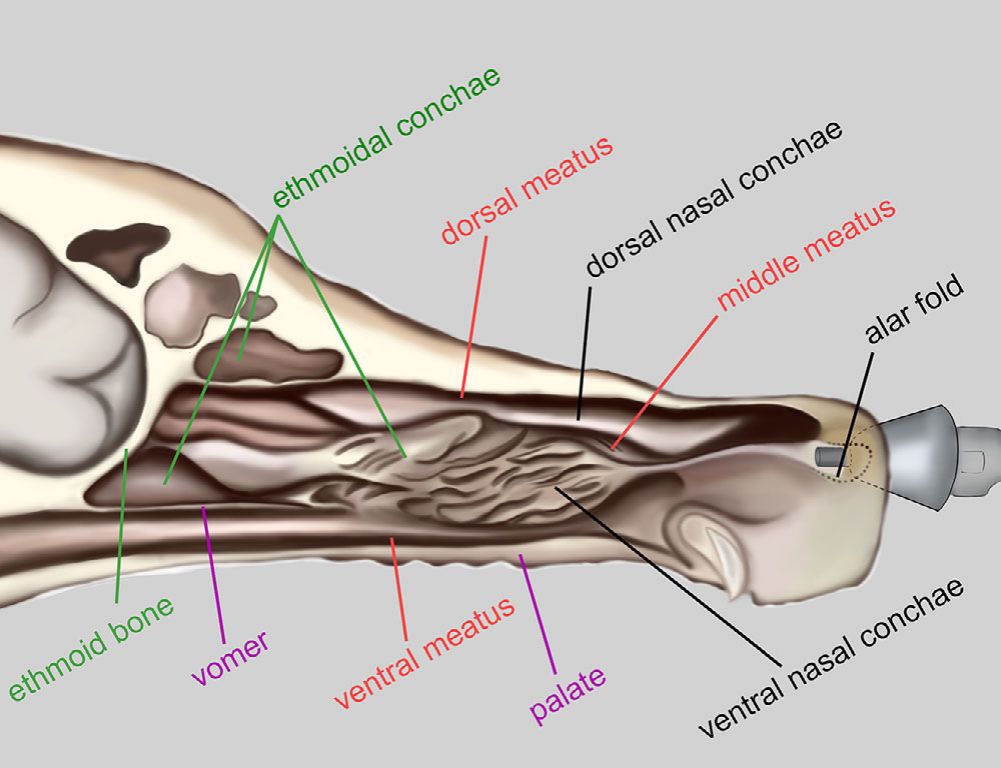
Schematic illustration showing the positioning of the MAD Nasal atomization device in a sagittal section of a mesocephalic large‐breed dog's head (nasal septum removed), emphasizing the importance of correct atomization angle. The nozzle should first be advanced medially beyond the alar fold and then oriented parallel to the rostral surface of the nasal bone, directed toward the ipsilateral eye, which is not visible in this view. The canine nasal cavity contains highly complex conchae that, while increasing the absorptive surface area, may restrict effective clearance and limit spray penetration into caudal regions. As a result, the ethmoidal conchae are unlikely to be reached, and deposition occurs primarily in the rostral nasal cavity. This schematic adapted and redrawn from Jafarbeglou et al. (2018).

Most veterinary studies have used the MAD Nasal (mucosal atomization device; Teleflex Medical), originally developed for humans (Charalambous et al. 2019; Degerfeld et al. 2023; Lin et al. 2024; Svoboda et al. 2023). According to the manufacturer, correct use in humans requires directing the nozzle toward the conchae and olfactory mucosa, with the soft conical plug fitting snugly in the nostril to prevent drug expulsion (MAD Nasal Device Procedure Guide 2025). A cadaveric study suggested that correct insertion and alignment toward the conchae are also critical in mesocephalic dogs. The nozzle should pass medially beyond the alar fold, then align parallel to the rostral surface of the nasal bone and nasal septum for optimal delivery to the nasal and ethmoidal conchae (Jafarbeglou et al. 2018). In practice, this corresponds approximately to the direction of the ipsilateral eye (author's note, Figure 3). However, as discussed earlier, effectively targeting the ethmoidal conchae in macrosmatic animals is nearly impossible (Gizurarson 2012, 1990).

**FIGURE 4:**
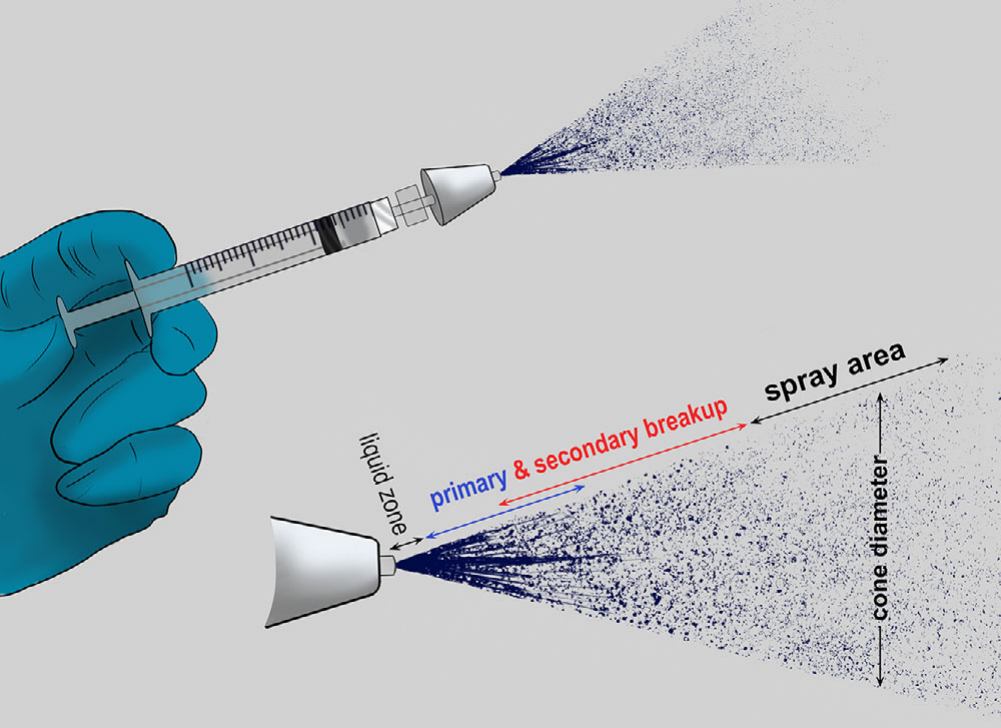
Schematic illustration of spray formation of the MAD Nasal atomization device. After exiting the nozzle, the liquid forms a jet that breaks into fine droplets at the breakup distance. The cone width and breakup length influence distribution and deposition of the drug. MAD Nasal produces a wide spray cone and a short breakup length, which requires sufficient intranasal clearance in both length and diameter. This schematic adapted and redrawn from Jafarbeglou et al. (2018).

An absent discussed aspect in veterinary reports is that spray formation follows fluid dynamic principles. In summary, after leaving the nozzle, the liquid exits as a coherent jet until the breakup length, where it fragments into droplets that expand into a cone‐shaped spray area (Figure 4). The spray angle and cone diameter determine dispersion and deposition efficiency, and the clearance needed for proper spray formation (Morita et al. 2025; Shrestha et al. 2023). If the nasal cavity is narrower than the spray cone diameter or lacks clearance, atomization fails and the liquid reverts to jets or drips. A supplemental video demonstrated that MAD Nasal produces a wide cone and short breakup length, requiring adequate space both in length and diameter (Jafarbeglou et al. 2018). These engineering requirements limit its use in species with narrow nasal passages such as cats, rabbits, brachycephalic and small‐breed dogs (Gizurarson 1990). Even in larger‐breed dogs, the high complexity of the nasal conchae may restrict effective clearance.

Brachycephalic dogs pose particular challenges due to shortened nasal passages and irregular nostrils, reducing IN efficiency compared with mesocephalic or dolichocephalic breeds (Ekenstedt et al. 2020; Gizurarson 1990). Successful application often requires holding the muzzle to secure the plug (Jafarbeglou, Marjani, Bakhshi‐Khanghah, et al. 2024; Lin et al. 2024), and poor fit is reported for small nostrils of some dogs (Charalambous et al. 2017). Consequently, many studies excluded small or brachycephalic dogs or limited experiments to mesocephalic breeds (Bustamante et al. 2024; Focken et al. 2024; Jafarbeglou, Marjani, Oghbaei, et al. 2024; Jafarbeglou, Marjani, Bakhshi‐Khanghah, et al. 2024; López‐Ramis et al. 2022).

In cats and rabbits, the challenge may be even greater. The device plug is substantially larger than their nostrils, making correct placement impossible. In a rabbit study, the 4.3 mm nozzle (MAD Nasal Device Procedure Guide 2025) had to be used without the plug, which caused expulsion and nasal runoff (Wei et al. 2022). Contrast medium in that study drained primarily into the ventral nasal meatus and pharynx (Wei et al. 2022), indicating a shift from atomization to dripping (Jafarbeglou et al. 2018). The manufacturer explicitly cautions that such dripping negates the benefits of atomization (MAD Nasal Device Procedure Guide 2025).

Clinically, these anatomical and engineering limitations explain why MAD Nasal often shows slower and weaker effects in cats and rabbits compared with IM injection (Degerfeld et al. 2023; Kaya and Yanmaz 2025), comparable to what reported for IND in dogs (Jafarbeglou, Marjani, Oghbaei, et al. 2024; Jafarbeglou and Marjani 2019). Thus, for small species, MAD Nasal may not offer significant advantages over the simpler, less costly IND technique. A clear understanding of spray dynamics and species‐specific nasal anatomy is therefore essential when adapting human‐designed atomizers for veterinary use.

#### 1.3.6 Other Reported Atomizers

Although MAD Nasal is the most common device, several alternatives have been used. A study employed a Wuxi NEST nasal atomizer (Liu et al. 2025), which has a similar design. Commercial single‐dose human sprays such as Narcan (Adapt Pharma; naloxone 4 mg, Barr et al. 2023; Essler et al. 2019; Wahler et al. 2019) and Neffy (ARS Pharma; epinephrine 1 mg, Sparapani et al. 2023), have also been used. Other studies described metered‐dose atomizers (Axiak et al. 2007; Henry et al. 1998). In two studies conducted on caribou and pigs, the MADgic laryngo‐tracheal atomization device (Teleflex Medical) was applied, functioning as a hybrid between INA and INC (Mathieu et al. 2022; Wiloch et al. 2024).

#### 1.3.7 Intranasal Catheterization (INC)

This method has been evaluated in rabbits (Freitag et al. 2022; Santangelo, Micieli, Marino, et al. 2016; Santangelo, Micieli, Mozzillo, et al. 2016; Weiland et al. 2017), cats (Yanmaz, Doğan, Okumuş, et al. 2017) and birds (Altundag et al. 2021; Net et al. 2019; Schaffer et al. 2017, Schaffer et al. 2016; Sha et al. 2022). Although it may reduce rostral runoff, advancing a foreign object into the nasal cavity can be highly uncomfortable and may trigger sneeze reflex (Dias et al. 2020; Songu and Cingi 2009). Furthermore, catheter should be advance into the ventral meatus toward nasopharynx (Freitag et al. 2022; Weiland et al. 2017), which can increase the risk of swallowing. Adverse outcomes have also been documented: in one rabbit study, mucosal trauma, bleeding and two deaths occurred, although the exact cause was not definitively established (Weiland et al. 2017).

#### 1.3.8 Swallowing, Nasal Runoff and Drug Loss

Among the factors influencing IN delivery, the administered volume is particularly critical (Bustamante et al. 2024; Hampton et al. 2021; Sha et al. 2022). Using low‐concentration formulations or diluting drugs for blinding purposes increases total volume. In some studies, the total volume exceeded the nasal cavity's capacity for a single administration, necessitating divided and repeated dosing (Wei, Chen, et al. 2023; Wei, Nakagawa, et al. 2023). The nasal cavity has limited retention capacity; when this threshold is exceeded, rostral or caudal runoff occurs, resulting in drug loss and reduced efficacy (Becker et al. 2021; Gao et al. 2020; Wei et al. 2022).

Swallowing is one of the most frequent adverse effects, reported in dogs (Bustamante et al. 2024; Jafarbeglou, Marjani, Bakhshi‐Khanghah, et al. 2024; López‐Ramis et al. 2022; Santangelo et al. 2019), pigs (Hampton et al. 2021; Rabelo et al. 2024) and rabbits (Santangelo, Micieli, Mozzillo, et al. 2016; Weiland et al. 2017), even in studies employing atomizers intended to minimize this problem. In dogs, swallowing occurred at volumes as low as 0.01 mL/kg per nostril (López‐Ramis et al. 2022), while several studies used volumes up to 0.04 mL/kg per nostril (Bustamante et al. 2024; Jafarbeglou, Marjani, Bakhshi‐Khanghah, et al. 2024; Jafarbeglou and Marjani 2019).

In pigs, administered volumes were substantially higher. For example, in a study combining azaperone, midazolam and ketamine, the total IN volume reached approximately 21 mL per animal (∼10 mL per nostril; ≈ 0.1 mL/kg). Audible gurgling sounds captured in the supplemental video confirms extensive runoff and swallowing (Rabelo et al. 2024). In another report on suckling piglets, an even higher total volume of 1.24 mL/kg (0.62 mL/kg per nostril) was described (Becker et al. 2021). By contrast, studies administering flunixin meglumine (2.2 mg/kg, 0.44 mL/kg), did not report swallowing (Lopez‐Soriano et al. 2023; Wiloch et al. 2024).

Concentrated formulations designed for IN use reduce required volume and improve retention. For example, Narcan delivers 4 mg naloxone in 0.1 mL (Essler et al. 2019), about 400 times more concentrated than standard injectable ampoules, which would require ∼10 mL to achieve the same dose (Wahler et al. 2019).

In the absence of nasal‐specific products, wildlife formulations may offer practical off‐label alternatives. These preparations are designed to achieve therapeutic effects with minimal volumes suitable for dart injection. The author has successfully used such concentrated wildlife formulations without complications. For instance, midazolam 50 mg/mL (≈ 10× standard concentration) was used in a ∼25‐kg dog for the management of status epilepticus, effectively reducing the required IN volume from 1.0 mL to 0.1 mL for a 0.2 mg/kg dose. Similar anecdotal use of concentrated wildlife‐formulated midazolam has been reported in hedgehogs (Doss and De Miguel Garcia 2022). To the best of the author's knowledge, concentrated wildlife formulations of other commonly used anaesthetic and sedative agents, such as ketamine, medetomidine, butorphanol, xylazine and atipamezole, are also commercially available. Nevertheless, further studies are warranted to confirm the safety of such preparations before widespread adoption.

Another approach to minimize swallowing is to divide the dose between both nostrils, thereby reducing per‐side volume and increasing the absorptive surface area (Gao et al. 2020). However, single‐nostril administration is faster, requires less restraint, may cause less distress and is often better tolerated (Jafarbeglou, Marjani, Bakhshi‐Khanghah, et al. 2024; Jafarbeglou and Marjani 2019). It also remains the standard approach for emergency, single‐use products (Barr et al. 2023; Sparapani et al. 2023).

#### 1.3.9 Nasopharyngeal Irritations and Related Adverse Reactions

Across nearly all reviewed species, the most common adverse reactions to IN administration are sneezing, reverse sneezing or sneezing‐like behaviours (Axiak et al. 2007; Ede et al. 2019; Enomoto et al. 2022; Marjani et al. 2015; Trevisan et al. 2016; Vlerick et al. 2020). These reactions are clinically relevant because they can compromise drug absorption and reduce efficacy (Breitenlechner et al. 2024; Jones et al. 2025; Yanmaz et al. 2022). As discussed earlier, nasal mucosa is highly sensitive, and both physical and chemical stimuli readily trigger the sneeze reflex (Songu and Cingi 2009); drug formulations, as chemical stimuli, are no exception.

In humans, the baseline nasal mucosal pH is approximately 6.3, which is slightly acidic. To minimize irritation, IN formulations are generally recommended to maintain a pH between 4.5 and 6.5 (Keller et al. 2022). Although species‐specific data on physiological nasal pH are limited, acidic injectable solutions such as midazolam are known to irritate the nasopharyngeal mucosa, causing pain or a burning sensation in humans (Antonio et al. 2011; Wermeling et al. 2006). Similar signs of nasopharyngeal irritation and discomfort have been reported in animals following IN administration of such compounds (Charalambous et al. 2017; Jafarbeglou, Marjani, Bakhshi‐Khanghah, et al. 2024; Mans et al. 2012; Schnellbacher et al. 2012; Vlerick et al. 2020).

Other physicochemical properties, including odour and taste, can also influence tolerability in both humans and animals (Antonio et al. 2011; Jafarbeglou, Marjani, Bakhshi‐Khanghah, et al. 2024). For instance, bitter taste has been reported after IN administration of midazolam and ketamine in paediatric patients (Reynolds et al. 2017; Wermeling et al. 2006). Related signs such as hypersalivation, drooling or oral frothing, reported in several animal studies (Bustamante et al. 2024; Dretchen et al. 2020a, 2020b; Jafarbeglou, Marjani, Bakhshi‐Khanghah, et al. 2024; Schnellbacher et al. 2012), may stem from similar taste‐mediated responses and can affect both absorption efficiency and tolerability. Conversely, compounds that are odourless, tasteless and less irritating, such as dexmedetomidine, tend to be better tolerated in human children and dogs (Cheng et al. 2024; Jafarbeglou, Marjani, Bakhshi‐Khanghah, et al. 2024; Talon et al. 2009).

#### 1.3.10 Other Reported Factors

The mucociliary clearance rate is a key determinant limiting drug absorption following IN administration (Gizurarson 2012). A few veterinary studies have evaluated IN gels designed to slow mucociliary clearance and reported superior outcomes compared with liquid formulations (Al‐Shebani 2011; Eagleson et al. 2012). By prolonging mucosal contact time, gels may enhance absorption efficiency (Eagleson et al. 2012).

#### 1.3.11 Is IN Administration Truly Less‐Stressful, Painless and Non‐Invasive?

In medical terminology, ‘noninvasive’ refers to procedures that do not involve penetration of the body or disruption of body tissue (dictionary.com). IN delivery is often described as non‐invasive and painless due to its needle‐free administration (Micieli, Santangelo, Napoleone, et al. 2017; Robertson and Eberhart 1994; Svoboda et al. 2023). However, as discussed earlier, adverse reactions, potential irritations and practical limitations have been consistently reported. In dogs and pigs, successful administration frequently requires muzzle or snout restraint (Jafarbeglou, Marjani, Bakhshi‐Khanghah, et al. 2024; Jafarbeglou and Marjani 2019; Lin et al. 2024; Lopez‐Soriano et al. 2023), which may be poorly tolerated by fearful, aggressive or anxious animals (Lin et al. 2024); ironically, the very patients most in need of sedation or chemical restraint (Axiak et al. 2007; Breitenlechner et al. 2024). Even in studies conducting on cooperative and non‐aggressive dogs, intolerance, defensive reactions and aggression have been reported (Bustamante et al. 2024; Essler et al. 2019; Jafarbeglou, Marjani, Bakhshi‐Khanghah, et al. 2024; López‐Ramis et al. 2022). Similar reactions are described in cats (Robertson et al. 2005), pigs (Breitenlechner et al. 2024), rabbits (Wei, Chen, et al. 2023, Wei, Nakagawa, et al. 2023; Weiland et al. 2017), reptiles (Emery et al. 2014; Sarri et al. 2025) and birds (Araghi et al. 2016).

IN administration can be performed smoothly in sedated or anesthetized animals (Barr et al. 2023; Dretchen et al. 2020b; Focken et al. 2024; Jafarbeglou, Marjani, Oghbaei, et al. 2024) or during ictal and postictal periods (Musulin et al. 2011). However, in conscious animals, it often requires prolonged or forceful restraint, with the head and neck immobilized, sometimes by two handlers (Jafarbeglou, Marjani, Bakhshi‐Khanghah, et al. 2024). Although shorter delivery times are generally better tolerated (Jafarbeglou, Marjani, Bakhshi‐Khanghah, et al. 2024), some procedures last over one minute, including pre‐ and post‐delivery restraint (Bustamante et al. 2024; Liu et al. 2025; Net et al. 2019; Zhuang et al. 2015). Such extended restraint can increase distress and complicate handling (Chastain and Vellios 2017), and often reflects larger administration volumes, which further elevate the risk of swallowing and reduced bioavailability.

Therefore, while IN administration may be less invasive physiologically, it is not necessarily less stressful in behavioural and welfare perspectives. Its overall value varies considerably between individuals and is influenced by species, temperament, arousal level and clinical context. On the other hand, despite certain advantages, IN delivery frequently yields comparable or inferior results to IM injection. Therefore, its use should be guided by critical questions: Is IN truly preferable in this case? Is it clinically necessary? Will the patient tolerate it better?

## 2 Conclusions

IN administration claimed as a valuable non‐invasive alternative to parenteral routes in veterinary medicine, offering rapid sedation, analgesia and emergency intervention. Evidence across multiple species supports its clinical potential, though efficacy remains variable due to anatomical differences, formulation issues and adverse reactions. Future progress requires establishing species‐specific case selection criteria, standardized dosing supported by pharmacokinetic and pharmacodynamic data, and safety studies assessing repeated or long‐term use. Developing concentrated, stable and well‐tolerated formulations may enhance practicality and consistency. Continued research should focus on balanced evaluation of benefits and limitations to ensure safe, effective and ethical clinical application.

## Author Contributions

**Majid Jafarbeglou**: conceptualization, methodology, investigation, visualization, data curation, formal analysis, writing – review and editing, writing – original draft.

## Funding

The author has nothing to report.

## Ethics Statement

The author confirms that the ethical policies of the journal, as noted on the journal's author guidelines page, have been adhered to. No ethical approval was required as this is a review article with no original research data.

## Conflicts of Interest

The author declares no conflicts of interest.

## Data Availability

The data that support the findings of this study are available from the corresponding author upon reasonable request.
